# Editorial Summary for the Special Issue “Prospective Studies on Harmful Cyanobacteria and Cyanotoxins”

**DOI:** 10.3390/toxins18090375

**Published:** 2026-08-31

**Authors:** Ekaterina N. Chernova, Sophia Barinova

**Affiliations:** 1 Scientific Research Centre for Ecological Safety, St. Petersburg Federal Research Center, Russian Academy of Sciences, St. Petersburg 197110, Russia; s3561389@yandex.ru; 2 Institute of Evolution, University of Haifa, Haifa 3498838, Israel

In recent decades, the problem of harmful algal blooms (HABs) in various water bodies has gained widespread recognition worldwide. Cyanobacteria are ubiquitous, including in extreme climates, and their metabolites, cyanotoxins, pose significant health risks. Despite the significant attention paid to harmful algal blooms (HABs), many knowledge gaps remain.

The aim of this Special Issue is to present new research findings on the geographic distribution of cyanotoxins in freshwater, their role in ecosystems, and methods for mitigating the impacts of HABs.

Published articles present research findings on the detection and quantification of cyanotoxins in various environments, using integrated methods (e.g., physicochemical, sensory, and molecular) to detect cyanotoxins and their producers.

This Special Issue presents studies on the distribution of cyanotoxins, starting with reports of cyanotoxin detection in water bodies in less studied regions, including results from monitoring drinking water bodies. Gabyshev et al. (Contribution 1) conducted a study on the presence of toxigenic *Microcystis* in the water and ice of a shallow eutrophic lake located in a continuous permafrost zone. *Microcystis* cells containing microcystin and the *mcy* genes were found to be present in the water column year-round, not only during the ice-free period but also under ice and within thick ice sheets. Kezlya et al. (Contribution 2) focused on the diversity and toxic potential of cyanobacteria in the natural and artificial aquatic environment of Moscow, Russia. A total of 20 cyanobacterial strains were isolated from the collected samples, namely representatives of *Anabaena*, *Aphanizomenon*, *Argonema*, *Dolichospermum*, *Microcystis*, and *Woronichinia*. Among them, the highest proportion (up to 28%) of MC-LR was recorded in two microcystin-producing strains of *Microcystis aeruginosa*.

A seasonal distribution of cyanobacteria in the plankton community also has severe implications for the availability of treated water. Piontek et al. (Contribution 3) summarized the previous data on the supply of consumer water in western Poland. The occurrence of HABs with the domination of *Aphanizomenon gracile* was revealed, and maximal microcystin concentrations were even above 50 mkg/L. The toxic properties of the waterbody samples were revealed using bioassays with the planarian *Dugesia tigrine*.

A number of published papers reported the information on mitigation techniques using algicides, ecosystem, and experimental approaches. Kuzikova et al. (Contribution 4) focused on a strain of antagonistic fungus *Penicillium* sp. isolated from a water sample from the Gulf of Finland, which demonstrated algicidal activity towards cyanobacteria (98–100%) and was able to degrade microcystin-LR. Transformation/decomposition products of the cyanotoxins formed were evaluated. The formation of less toxic intermediates was proved using *Daphnia magna* as a bioindicator. It was concluded that ascomycete fungi could be used not only for cyanoHABs control but also for detoxification of water bodies. Kurbatova et al. (Contribution 5) investigated the effects of an algicide derived from macrophyte metabolites, namely mixtures of gallic, tetradecanoic, heptanoic, and octanoic acids. Two types of microcosms have been created in the study: simple (consisting of microalgae, cyanobacteria, and zooplankton) and complex (microalgae, cyanobacteria, zooplankton, and planktivorous fish). It was found that the algicide properties of macrophyte metabolites differed depending on the ecosystem type.

During algal blooms, variable concentrations of different structural forms of microcystins were recorded, partitioning between the dissolved and particulate phases, influencing the toxicity of water samples. Pearce et al. (Contribution 6) assessed microcystin dynamics during active growth and biomass degradation using incubation experiments with six phytoplankton samples collected from the Chowan River. Microcystins were released from cells at an exponential rate, but extracellular dissolved toxins persisted in water for over 100 days. The congener composition of the intracellular and dissolved fractions changed between phases and over time, altering exposure pathways (e.g., drinking water contamination or contamination via the food chain). This study provides useful information for monitoring strategies and assists in assessing the risk of microcystin exposure during algal blooms.

During harmful algal blooms, cyanobacterial toxins, including microcystins, are released into water, posing a public health threat. Rife et al. (Contribution 7) assessed the validity of using self-reported symptom data in epidemiological studies related to harmful algal blooms. In the study, statistical methods were used to compare symptom reports during active blooms and in the absence of blooms.

Toxicity assessment was conducted using test species from tropical and subtropical water bodies. Zamora-Barrios et al. (Contribution 8) assessed the effects of crude cyanobacterial extracts on three common native species (*Daphnia laevis*, *Ceriodaphnia dubia*, and *Simocephalus vetulus*). The results were compared with the tolerance of the common test organism Daphnia magna based on acute and chronic bioassays. This study shows that the non-model species *Simocephalus vetulus* was highly susceptible to crude cyanobacterial extracts, making it promising for toxicity assessment in tropical ecosystems. The study demonstrates the value of incorporating non-model species into ecotoxicological assessments for use in local environmental monitoring.

Thus, the series of publications in the Special Issue covers the impacts of toxic algal blooms in different regions and in both natural and experimental systems, expanding the awareness of the scientific community and those involved in decision-making in order to preserve natural diversity and the high quality of natural waters used by humans.

